# Genetic Variation and Haplotype Structure of *CFAP299* and *PRDM8* Associated with Fiber Yield and Hair Length Traits in Tianzhu White Yak

**DOI:** 10.3390/life16091559

**Published:** 2026-09-17

**Authors:** Yicheng Liu, Xuedong Qi, Yongfu La, Xiaoming Ma, Wenxue Luo, Wenwen Ren, Guowu Yang, Zhenyu Zhang, Min Chu, Xiaoyun Wu, Xian Guo, Shaobin Li, Wanzhen Qi, Chunnian Liang

**Affiliations:** 1 Key Laboratory of Yak Breeding Engineering of Gansu Province, Lanzhou Institute of Husbandry and Pharmaceutical Sciences of Chinese Academy of Agricultural Sciences, Lanzhou 730050, China; 19539592141@163.com (Y.L.);; 2 Key Laboratory of Animal Genetics and Breeding on Tibetan Plateau, Ministry of Agriculture and Rural Affairs, Lanzhou 730050, China; 3 College of Animal Science and Technology, Gansu Agricultural University, Lanzhou 730070, China; 4 Tianzhu Tibetan Autonomous County Animal Husbandry Technology Extension Station, Tianzhu 733200, China

**Keywords:** Tianzhu White Yak, *CFAP299* and *PRDM8*, fiber yield, hair length traits, SNPs, haplotype

## Abstract

Fiber yield and hair length are important economic traits in Tianzhu White Yak. However, the genetic variants associated with fiber production performance in this breed remain insufficiently characterized. This study aimed to investigate the associations of single nucleotide polymorphisms (SNPs) in the *CFAP299* and *PRDM8* genes with fiber yield and hair length traits in Tianzhu White Yak and to identify potential molecular markers for marker-assisted selection. A total of 759 Tianzhu White Yaks were included in this study. Genomic DNA was extracted from blood samples, SNP detection and genotyping were performed using GATK (v. 4.2.6.1), and candidate SNPs were validated by PCR amplification and Sanger sequencing. Five SNPs in *CFAP299* and three SNPs in *PRDM8* were selected for analyses of genetic diversity, Hardy–Weinberg equilibrium, linkage disequilibrium, haplotype structure, and associations with phenotypic traits. All eight loci displayed three genotypes. No significant departure from Hardy–Weinberg equilibrium was detected at any locus (*p* > 0.05), and PIC values of 0.25–0.50 indicated moderate polymorphism. After adjustment for sex and age and FDR correction, all eight SNPs remained significantly associated with head hair length (HL) and fiber yield (FY), while several loci were also significantly associated with body-side hair length (BSL) and back hair length (BL) (*q* < 0.05). Individuals carrying mutant homozygous genotypes generally exhibited higher phenotypic values for the corresponding traits, whereas heterozygous genotypes generally showed intermediate values between the two homozygous genotypes. Linkage disequilibrium and haplotype analyses revealed that the eight SNPs formed three haplotype blocks. Haplotype combinations in Blocks 1 and 3 were significantly associated with variation in HL, FY, and SL, whereas no significant associations were detected for Block 2. Overall, SNPs and haplotype combinations in *CFAP299* and *PRDM8* were associated with variation in fiber yield and hair length traits in Tianzhu White Yak. These findings support *CFAP299* and *PRDM8* as candidate genes associated with fiber-related traits and provide potential molecular markers for further validation and marker-assisted selection.

## 1. Introduction

Yaks (*Bos grunniens*) inhabit harsh high-altitude environments characterized by low oxygen availability, large diurnal temperature fluctuations, low temperatures, and limited pasture resources. Through long-term natural selection, they have developed a strong ability to tolerate coarse feed, hunger, and cold. Their adaptability to these extreme environmental conditions makes them a valuable genetic resource [1]. Yaks provide local residents with economically valuable products such as milk, hides, meat, and fuel [2], while also serving as a means of transportation, thereby offering convenience to the local population [3]. The Tianzhu White Yak is a local yak population native to Gansu Province and is characterized by its pure white coat. This population is of particular interest for studies of fiber yield and hair length and represents a valuable genetic resource for the breeding of long-haired and standard-haired subpopulations [4]. Tianzhu White Yaks are also characterized by their large, well-proportioned bodies, well-developed musculature, tolerance to coarse feed, and prominent withers. Their meat, milk, hair, fleece, bones, and hides are economically important products [5]. In addition to the economic benefits derived from meat and milk production, the fleece of Tianzhu White Yak has considerable utilization value [6]. The coat of the Tianzhu White Yak is valued for its distinctive appearance and high-quality down and is widely used in ethnic clothing, carpet manufacturing, and cold-weather protective products [7]. In addition to its economic and cultural importance, the coat plays an important role in adaptation to cold environments. It provides effective thermal insulation, reduces heat loss, and thereby contributes to the survival of Tianzhu White Yaks under extreme climatic conditions [8]. In recent years, increasing research attention has focused on the coat characteristics and hair follicle biology of Tianzhu White Yak. These studies have included transcriptomic and single-cell transcriptomic analyses of hair follicle anagen and telogen phases [9], whole-genome CNV analyses of long-haired and standard-haired Tianzhu White Yaks [10], and studies on the molecular regulation of the hair follicle cycle [7].

Fiber production is influenced by a combination of factors, including genetics, nutrition, and the environment, with genetic factors providing the foundation for the improvement of fiber production traits [11]. Previous studies have shown that wool and down traits generally exhibit moderate to high heritability. Antagonistic genetic correlations may exist between average fiber diameter and other fiber traits, such as down yield, down weight, and down length, indicating considerable potential for genetic improvement of these traits [12]. Mizuno et al. [13] reported that a 1 bp deletion in the *FGF5* gene of Syrian hamsters was associated with a long-haired phenotype. Legrand et al. [14] sequenced the *FGF5* gene in 35 long-haired donkeys, 67 short-haired donkeys, and 131 short-haired ponies and identified five missense mutations and a frameshift mutation, indicating allelic heterogeneity underlying the long-haired phenotype in donkeys. Previous studies have also shown that candidate regions associated with the long-haired trait in Tianzhu White Yak are mainly concentrated on chromosome 6, and multiple candidate genes related to hair length and fiber production performance, such as *FGF5*, *ATP8A1*, and *SLC30A9*, have been identified [4]. Zhou et al. [15] investigated transcriptional differences between long-haired Tianzhu White Yak (LHY) and normal-haired yak (NHY) and identified differential expression of genes including *BMP4* and *KRT2* in forehead skin, and *BMP1*, *KRT1*, and *FGF5* were present in shoulder skin.

The cilia and flagella associated protein 299 (*CFAP299*) gene, also known as chromosome 4 open reading frame 22, is located downstream of the *FGF5* gene. In a study of selection signatures in Tianzhu White Yak, the *CFAP299* and *FGF5* genes were identified within selection hotspot regions associated with long-haired and normal-haired Tianzhu White Yak, respectively, on chromosome 6. Furthermore, GO and KEGG pathway analyses of the genomic region containing *CFAP299*, including enrichment of the MAPK signaling pathway, suggested that *CFAP299* may be a candidate gene associated with coat length in Tianzhu White Yak [4]. Lehman et al. [16] reported that primary cilia are involved in hair follicle keratinocyte differentiation and hair follicle morphogenesis. Since *CFAP299* belongs to the family of cilia- and flagella-associated proteins, it may be involved in hair follicle development and hair growth, although direct evidence for such a role remains limited. In addition, phylogenetic analyses have identified the *CFAP299* gene as a cilia-associated gene, but its specific role in ciliogenesis has not yet been fully characterized [17]. The *PRDM8* gene encodes PRDM-BF1 and RIZ homology domain-containing protein 8, a member of the PR/SET domain family [18]. PRDM family proteins contain an N-terminal PR domain and multiple zinc finger domains and are involved in developmental regulation, cell fate determination, and transcriptional regulation [19]. Previous studies have shown that *PRDM8* can form a transcriptional repressor complex with Bhlhb5 and participate in neural circuit assembly, supporting its role as a transcriptional regulator [20]. Pallotti et al. [21] reported that *PRDM8* co-localizes with genes such as *FGF5* and *ANTXR2* within genomic regions associated with fiber traits, suggesting that these regions may have been selected for both productive traits and natural adaptation to harsh environments. Although studies linking *PRDM8* to mammalian coat traits remain limited, our previous GWAS identified *PRDM8* as a candidate gene associated with coat-related traits in Tianzhu White Yak [6]. *PRDM8* has also been investigated in cancer research. In hepatocellular carcinoma, *PRDM8* was reported to act as a tumor suppressor through negative regulation of NAP1L1 and suppression of the PI3K/AKT/mTOR signaling pathway [22].

SNP-based approaches have been widely applied in livestock genetic studies to identify genomic regions, candidate genes, and molecular markers associated with economically important traits, particularly through genome-wide association studies (GWAS), and have provided valuable information for marker-assisted selection [23]. Kunene et al. [24] used high-density SNP genotype data to investigate the genetic basis of coat color variation and patterns in South African Nguni cattle. SNPs have also been used to identify candidate genes associated with coat length traits in long-haired and standard-haired Tianzhu White Yaks [4]. However, research on genetic variants associated with fiber production traits in yaks remains limited, and further investigation is needed to identify candidate genes and potential molecular markers associated with these traits. In our previous GWAS in the same 759 Tianzhu White Yaks, *CFAP299* and *PRDM8* were identified as candidate genes associated with fiber yield and hair length traits [6]. Based on these results, the present study selected eight SNPs in these two genes for Sanger sequencing validation and further investigated their genetic diversity, linkage disequilibrium, haplotype structure, and associations with fiber yield and hair length traits at both the single-SNP and haplotype levels. The aim was to further evaluate these loci as candidate molecular markers associated with fiber production traits in Tianzhu White Yak and to provide a basis for future marker-assisted selection.

## 2. Materials and Methods

### 2.1. Experimental Materials

Animals were sampled in mid-June 2025 from Xiamao’ergou Village, Dachai’gou Town, Tianzhu Tibetan Autonomous County, Wuwei City, Gansu Province. A total of 759 healthy Tianzhu White Yaks originating from the same breeding population were included. Phenotypic measurements were obtained in vivo by the same research team. These 759 animals were also used in our previous GWAS of fiber yield and hair length traits [6]. In the present study, selected SNPs in *CFAP299* and *PRDM8* identified from the previous GWAS were further investigated. For each animal, every phenotype was recorded twice, and the mean of the two measurements was used for statistical analysis. The animals ranged from 1 to 10 years of age, had similar body conditions, and were maintained under comparable management conditions. Of the 759 animals, 564 were female and 195 were male. Approximately 5 mL of whole blood was obtained from each animal by venipuncture and collected into anticoagulant vacuum tubes. Samples were gently mixed immediately after collection and stored at −20 °C until DNA extraction.

### 2.2. Phenotypic Data Collection and DNA Extraction from Blood Samples

Phenotypic records included skirt hair length, body side hair length, head hair length, back hair length, and fiber yield. Hair length traits were recorded with a ruler, whereas fiber yield was determined using an electronic scale (Shanghai Puchun Measure Instrument Co., Ltd., Shanghai, China). Trait distributions were inspected using descriptive statistics and graphical assessment. Although several traits showed minor departures from normality, no pronounced skewness was observed; therefore, the raw phenotypic values were used directly in subsequent analyses. Genomic DNA was extracted from blood samples according to the manufacturer’s instructions using the TIANamp Genomic DNA Kit (TIANGEN Biotech (Beijing) Co., Ltd., Beijing, China). DNA purity was assessed based on the OD260/280 ratio, and DNA concentration was measured using a Fluo-100 Fluorometer (Hangzhou Allsheng Instruments Co., Ltd., Hangzhou, China). DNA concentrations ranged from 70 to 145 ng/μL.

### 2.3. Genotyping

Whole-genome resequencing was conducted for DNA samples that met the quality requirements using the DNBSEQ-T7 platform (MGI Tech Co., Ltd., Shenzhen, China). SOAPnuke (v. 2.2.1) was applied to remove low-quality reads and obtain clean sequencing data [25]. Clean reads were mapped against the Bosgru_v3.0 yak reference genome with BWA (v. 0.7.17) [26], after which SAMtools (v. 1.9) was used to generate coordinate-sorted BAM files [27]. Alignment quality was evaluated with the BamQC module implemented in Qualimap2 (v. 2.2.2-dev) [28]. Variant discovery and genotype calling were carried out with GATK (v. 4.2.6.1) [29], and SnpEff (v. 5.0) was used for SNP annotation [30]. The SNP dataset was further filtered using Plink (v.1.90). SNPs with a genotype missing rate > 10%, a minor allele frequency (MAF) < 0.01, or a Hardy–Weinberg equilibrium *p*-value < 1 × 10^−5^ were excluded. Individuals with a genotype missing rate > 30% were also removed.

### 2.4. SNP Site Validation and Primer Design

Based on our previous GWAS results [6], eight SNPs in *CFAP299* and *PRDM8* that passed the above quality control criteria were selected for further validation. The sequences encompassing the SNP loci g.24886108 A > T, g.24928028 C > T, g.25200739 G > A, g.25210441 C > T, g.25237572 A > C, g.25499166 G > A, g.25571255 G > T, and g.25622784 C > T were amplified to validate the genotyping results obtained from whole-genome resequencing. Genomic regions flanking each SNP were obtained from the Bosgru_v3.0 yak reference genome (GCA_005887515.1). Primer pairs spanning the target loci were designed with NCBI Primer-BLAST (https://www.ncbi.nlm.nih.gov/tools/primer-blast/, accessed on 18 March 2026) and were subsequently synthesized by Shanghai Sangon Biotech Co., Ltd. (Shanghai, China). The resulting PCR products were used for Sanger sequencing to verify the genotypes obtained from whole-genome resequencing. All eight selected SNPs were located on chromosome 6. Primer sequences, expected amplicon sizes, and annealing temperatures are provided in Table 1.

### 2.5. PCR Amplification and Sequencing

PCR amplification was performed for the eight SNP loci described above using genomic DNA (gDNA) extracted from Tianzhu White Yak blood samples as the template. The total reaction volume was 25 μL, consisting of 6.5 μL dd H_2_O, 4 μL genomic DNA (10 ng/μL), 1 μL each of the forward and reverse primers (10 μmol/L), and 12.5 μL TaKaRa Taq™ Version 2.0 plus dye (Takara Biotechnology (Dalian) Co., Ltd., Dalian, China). The PCR conditions were as follows: initial denaturation at 95 °C for 3 min; 35 cycles of denaturation at 95 °C for 30 s, annealing at 59 °C for 30 s, and extension at 72 °C for 20 s; followed by a final extension at 72 °C for 5 min. The reactions were then held at 4 °C. The amplification products were analyzed by 1% agarose gel electrophoresis (110 V, 40 min) to confirm that the amplified fragments were of the expected size. The PCR products, together with the forward and reverse primers, were then sent to Shanghai Sangon Biotech Co., Ltd. (Shanghai, China) for Sanger sequencing to validate the target SNP genotypes.

### 2.6. Statistical Analysis

MEGA 11.0 software was used to inspect sequencing results and perform genotype identification, and the obtained data were organized using Excel 2019. Genetic diversity parameters, including minor allele frequency (MAF), expected heterozygosity (He), effective number of alleles (Ne), polymorphism information content (PIC), and allele frequencies, were calculated using Excel functions. Hardy–Weinberg equilibrium (HWE) tests were performed for each SNP locus.

The associations between SNP genotypes and hair-related traits were analyzed using general linear models (GLMs) implemented in R (v.4.5.1). To account for the potential effects of sex and age on hair production traits, genotype, sex, and age were included as fixed effects in the model:

Yi = μ + Gi + Sexi + Agei + ei

where Yi represents the phenotypic value of the ith individual, μ represents the overall population mean, Gi represents the effect of SNP genotype, Sexi and Agei represent the fixed effects of sex and age, respectively, and ei represents the random residual error. The association results for the eight SNPs were obtained using this model. Least-squares means (LS-means) and standard errors (SE) were estimated for each genotype using the GLM, and multiple comparisons were performed using Tukey’s test.

Linkage disequilibrium (LD) analysis among SNP loci in the *CFAP299* and *PRDM8* genes was performed using Haploview software (v4.2). Haplotype blocks were constructed based on linkage disequilibrium parameters (D′ and r^2^). Strongly linked SNPs were further analyzed using PHASE software (v2.0) to infer haplotypes and diplotype combinations. Rare haplotype combinations with frequencies < 1% or insufficient sample sizes were excluded from subsequent association analyses. The association between haplotype combinations and fiber traits was analyzed using a GLM in which haplotype combination, sex, and age were included as fixed effects:

Yi = μ + Hi + Sexi + Agei + ei

where Hi represents the effect of haplotype combination. Thus, both the eight SNPs and haplotype association analyses were adjusted for sex and age. The results were presented as LS-means ± SE.

For all SNP–trait association analyses, multiple testing correction was performed using the Benjamini–Hochberg false discovery rate (FDR) method, and FDR-adjusted q-values < 0.05 were considered statistically significant.

## 3. Results

### 3.1. Genotyping Results and Genetic Diversity Analysis of the CFAP299 and PRDM8 Genes in Tianzhu White Yak

**Figure 1:**
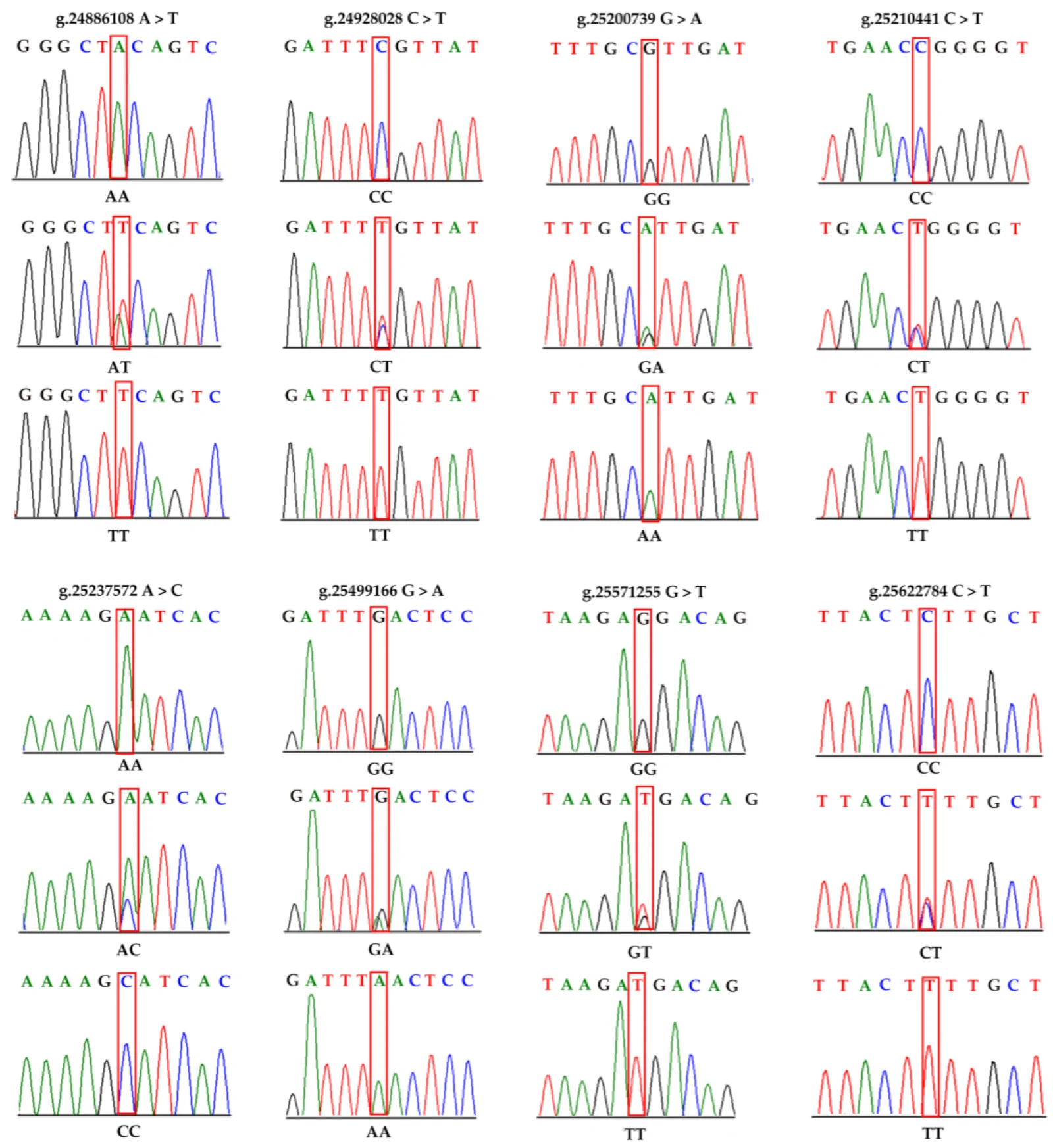
Sanger sequencing chromatograms of eight SNP loci in *CFAP299* and *PRDM8*. Nucleotide bases are color-coded as follows: A, green; T, red; C, blue; and G, black. Red boxes indicate the target SNP positions.

As shown in Figure 1, Sanger sequencing was performed to validate the PCR amplification products of eight candidate SNP loci in the *CFAP299* and *PRDM8* genes. The results showed that these eight loci contained A > T, C > T, G > A, C > T, A > C, G > A, G > T, and C > T base substitutions, respectively, and three genotypes were detected at each SNP locus, indicating that the sequencing results were consistent with the genotyping results.

**Figure 2:**
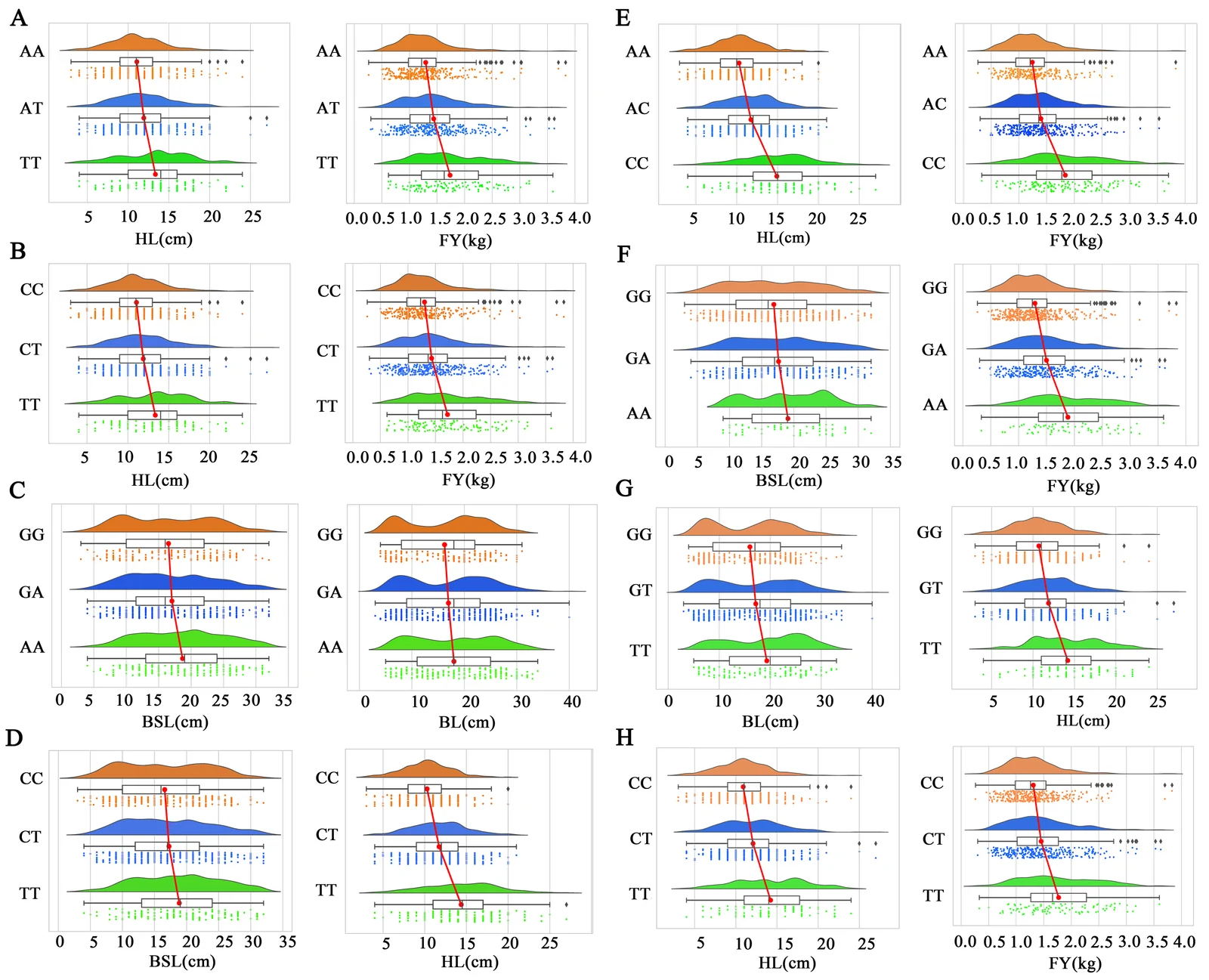
Distribution of the eight SNP genotypes in relation to major hair length traits and fiber yield in Tianzhu White Yak. (**A**) g.24886108 A > T; (**B**) g.24928028 C > T; (**C**) g.25200739 G > A; (**D**) g.25210441 C > T; (**E**) g.25237572 A > C; (**F**) g.25499166 G > A; (**G**) g.25571255 G > T; and (**H**) g.25622784 C > T. BSL, body-side hair length; HL, head hair length; BL, back hair length; FY, fiber yield. The density curves show the distribution of phenotypic values for each genotype, the boxplots summarize the distribution within each genotype, and the colored dots represent individual phenotypic observations. Black diamonds indicate outlying observations identified by the boxplots. Red points connected by red lines indicate the mean phenotypic values of the three genotypes at each SNP locus.

The descriptive statistics of fiber yield and hair length traits in 759 yaks are shown in Table 2. Genotype frequency, allele frequency, polymorphism information content, heterozygosity, and Hardy–Weinberg equilibrium were calculated, and the detailed results are presented in Table 3. All eight SNP loci exhibited three genotypes in the Tianzhu White Yak population. Figure 2 shows the distribution of the three genotypes of each SNP locus across the hair length traits and fiber yield of Tianzhu White Yak. Overall, the three genotypes were well represented at most loci. For the five SNP loci in *CFAP299*, mutant homozygous individuals generally showed higher mean values of BSL, BL, HL, and FY than wild-type homozygous individuals. Heterozygous genotypes generally showed intermediate phenotypic values between the two homozygous genotypes. A similar pattern was observed for the three SNP loci in *PRDM8*, with mutant homozygous individuals generally showing higher mean values of BSL, HL, BL, and FY than wild-type homozygous individuals.

Among the five SNP loci in the *CFAP299* gene, the AT, CT, GA, CT, and AC genotypes had the highest genotype frequencies, with values of 0.436, 0.455, 0.493, 0.493, and 0.476, respectively, indicating that heterozygous genotypes were predominant at these loci. The A allele showed the highest frequency at g.24886108 A > T and g.25237572 A > C, while the C allele showed the highest frequency at g.24928028 C > T and g.25210441 C > T, indicating that the wild-type alleles were predominant at these four loci. At g.25200739 G > A, the A allele had the highest frequency. The PIC values of these five loci ranged from 0.25 to 0.50, indicating moderate polymorphism, and all five loci conformed to HWE (*p* > 0.05). Among the three SNP loci in the *PRDM8* gene, the GG, GT, and CC genotypes had the highest genotype frequencies, at 0.506, 0.484, and 0.455, respectively. The heterozygous GT genotype was the most frequent at g.25571255 G > T. The major allele was G at both g.25499166 G > A and g.25571255 G > T, while T was the major allele at g.25622784 C > T, indicating that the wild-type alleles were predominant at these three loci. The PIC values of these three loci were 0.325, 0.364, and 0.346, respectively, indicating moderate polymorphism (0.25 < PIC < 0.50). All three loci conformed to Hardy–Weinberg equilibrium (*p* > 0.05).

### 3.2. Association Analysis of Different Genotypes at SNP Loci of the CFAP299 and PRDM8 Genes in Tianzhu White Yak with Fiber Yield and Hair Length Traits

Association analysis between individual SNP genotypes and fiber yield and hair length traits in Tianzhu White Yak was performed using a GLM incorporating genotype, sex, and age as fixed effects. The association results for five SNP loci in the *CFAP299* gene, including g.24886108 A > T, g.24928028 C > T, g.25200739 G > A, g.25210441 C > T, and g.25237572 A > C, are presented in Table 4. After FDR correction, g.24886108 A > T and g.24928028 C > T remained significantly associated with HL and FY (*q* < 0.05). The other three loci, g.25200739 G > A, g.25210441 C > T, and g.25237572 A > C, remained significantly associated with BSL, HL, BL, and FY (*q* < 0.05). Across the five loci, individuals carrying mutant homozygous genotypes generally exhibited higher HL and FY values than those carrying wild-type homozygous genotypes. Heterozygous genotypes generally showed intermediate phenotypic values between the two homozygous genotypes. At g.25200739 G > A, both the GA and AA genotypes showed significantly higher BL values than the GG genotype. These results indicate that *CFAP299* polymorphisms are associated with variation in hair-related traits and may serve as candidate genetic markers for fiber-related traits in Tianzhu White Yak.

Association analysis was also performed between three SNP loci in the PRDM8 gene, including g.25499166 G > A, g.25571255 G > T, and g.25622784 C > T, and fiber-related traits in Tianzhu White Yak. The results are presented in Table 5. After FDR correction, all three loci remained significantly associated with HL and FY (*q* < 0.05). The g.25499166 G > A locus was also significantly associated with BL, the g.25571255 G > T locus was significantly associated with BSL and BL, and the g.25622784 C > T locus was significantly associated with BL (*q* < 0.05). At g.25499166 G > A, individuals with the AA genotype showed significantly higher HL, BL, and FY values than those with the GG genotype. At g.25571255 G > T, individuals with the TT genotype showed significantly higher BSL, HL, BL, and FY values than those with the GG genotype. At g.25622784 C > T, individuals with the TT genotype showed significantly higher HL and FY values than those with the CC genotype. These results indicate that *PRDM8* polymorphisms are associated with variation in fiber-related traits in Tianzhu White Yak and may serve as candidate genetic markers for these traits.

### 3.3. Analysis of Linkage Disequilibrium and Haplotype Analysis of SNP Locations in the CFAP299 and PRDM8 Genes of Tianzhu White Yak

**Figure 3:**
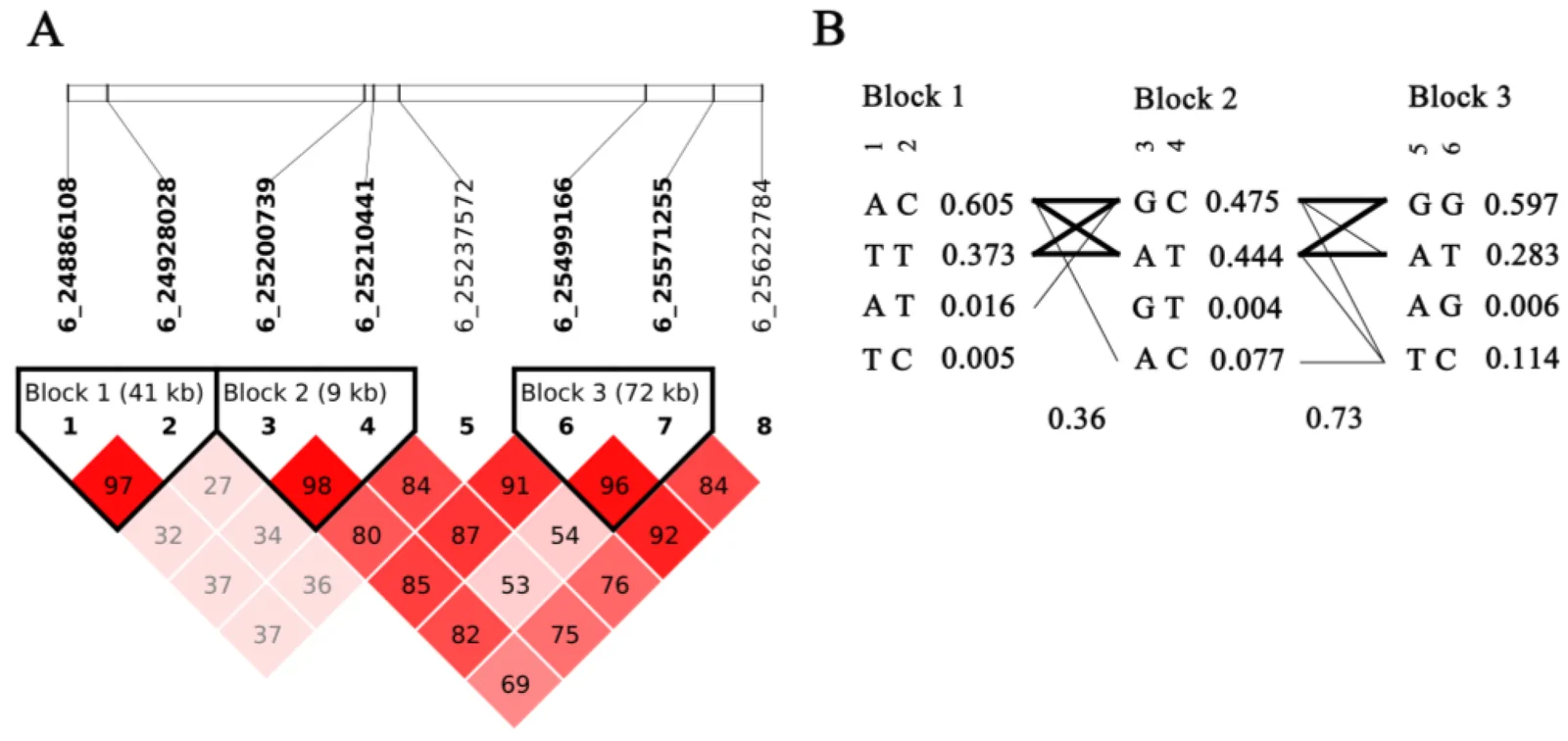
Linkage disequilibrium and haplotype structure analysis of eight SNPs in Tianzhu White Yak. (**A**) Linkage disequilibrium analysis of SNPs. The numbers within diamonds indicate the percentage of D′ values between loci, and darker colors represent stronger linkage disequilibrium. (**B**) Haplotype block analysis. D′ = 1 indicates complete linkage disequilibrium, whereas D′ = 0 indicates no linkage disequilibrium or linkage equilibrium.

The eight SNPs that conformed to Hardy–Weinberg equilibrium were further analyzed. As shown in Figure 3, the eight SNP loci formed three haplotype blocks. Block 1 consisted of g.24886108 A > T and g.24928028 C > T, with a block length of 41 kb; Block 2 consisted of g.25200739 G > A and g.25210441 C > T, with a block length of 9 kb; and Block 3 consisted of g.25499166 G > A and g.25571255 G > T, with a block length of 72 kb. Figure 3A shows that the SNPs within each block exhibited strong linkage disequilibrium (LD). The D′ values expressed as percentages within Blocks 1, 2, and 3 were 97, 98, and 96, respectively, indicating strong linkage disequilibrium among the SNPs within each block. Figure 3B shows that AC and TT were the major haplotypes in Block 1, with frequencies of 0.605 and 0.373, respectively; GC and AT were the major haplotypes in Block 2, with frequencies of 0.475 and 0.444, respectively; and GG, AT, and TC were the major haplotypes in Block 3, with frequencies of 0.597, 0.283, and 0.114, respectively. A total of 24 haplotype combinations were identified across the three blocks (Table 6), among which eight combinations had fewer than 10 individuals. Therefore, haplotype combinations represented by fewer than 10 individuals were excluded from subsequent association analyses.

### 3.4. Association Analysis of Different Haplotype Combinations in the CFAP299 and PRDM8 Genes with Hair Length Traits and Fiber Yield in Tianzhu White Yak

**Figure 4:**
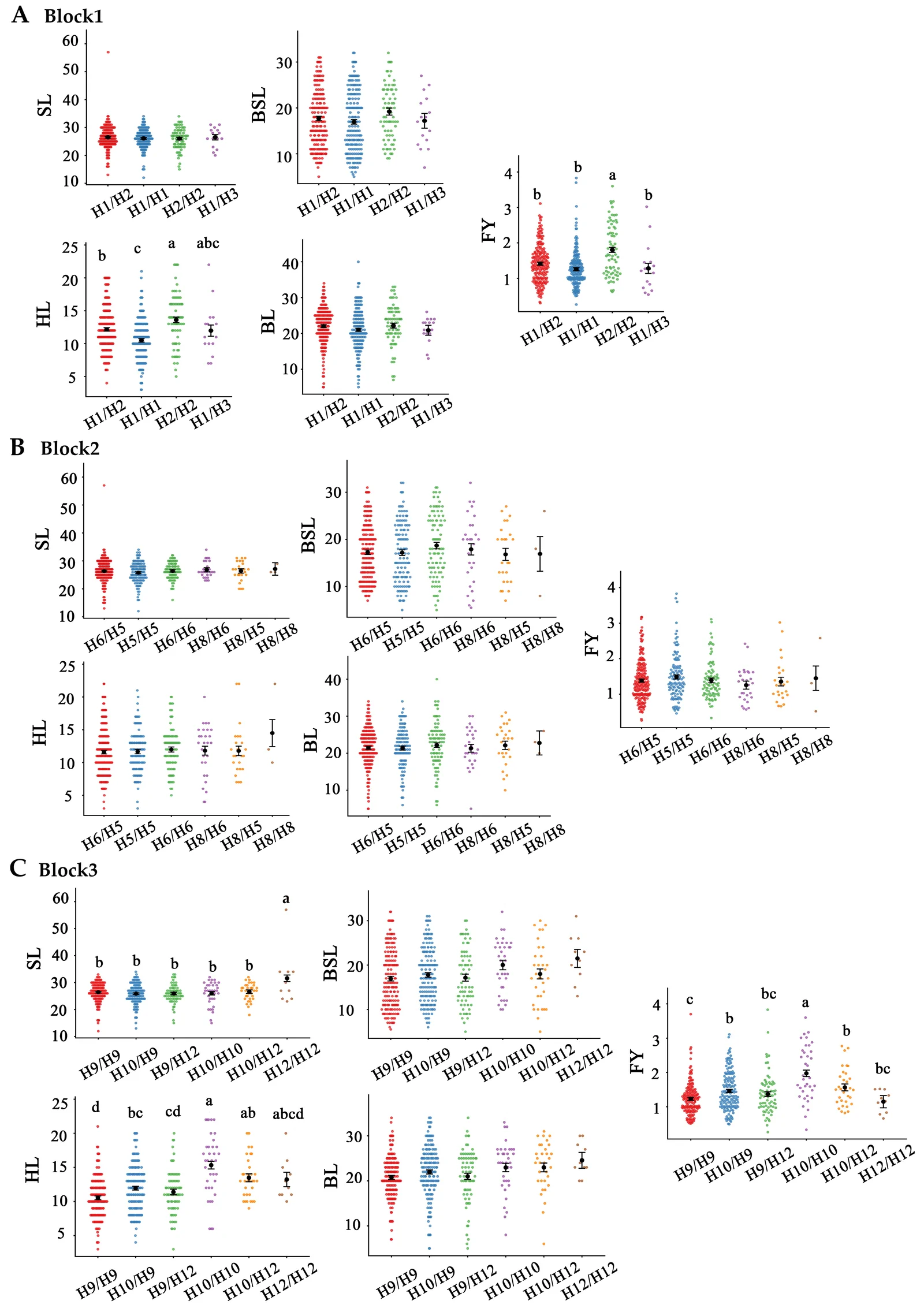
Association between haplotype combinations in *CFAP299* and *PRDM8* and hair length traits and fiber yield in Tianzhu White Yak. (**A**) Block 1; (**B**) Block 2; and (**C**) Block 3. SL, skirt hair length; BSL, body-side hair length; HL, head hair length; BL, back hair length; FY, fiber yield. Colored dots represent individual phenotypic values, and black circles with error bars represent least-squares means ± standard error (LS-means ± SE) estimated from the GLM adjusted for sex and age. Different lowercase letters above haplotype combinations indicate significant differences according to Tukey’s multiple-comparison test (*p* < 0.05), whereas haplotype combinations sharing at least one lowercase letter do not differ significantly. For traits without lowercase letters, no significant differences among haplotype combinations were detected (*p* > 0.05).

Associations between haplotype combinations of the three haplotype blocks and fiber yield and hair length traits in Tianzhu White Yak were evaluated. The results are shown in Figure 4 and Supplementary Table S1. In Block 1, no significant differences were observed among haplotype combinations for SL, BSL, and BL (*p* > 0.05), whereas significant differences were detected for HL and FY (*p* < 0.05). The H2/H2 combination showed significantly higher HL than the H1/H2 and H1/H1 combinations, and its FY was significantly higher than those of the H1/H2, H1/H1, and H1/H3 combinations. In Block 2, no significant differences were observed among haplotype combinations for SL, BSL, HL, BL, or FY (*p* > 0.05), indicating that Block 2 haplotype combinations were not significantly associated with these traits. In Block 3, significant differences were detected among haplotype combinations for SL, HL, and FY (*p* < 0.05), whereas no significant differences were observed for BSL and BL (*p* > 0.05). The H12/H12 combination showed significantly higher SL than the other combinations. For HL, the H10/H10 combination showed significantly higher values than the H9/H9, H10/H9, and H9/H12 combinations. For FY, the H10/H10 combination was significantly higher than the other haplotype combinations. These results indicate that haplotype combinations in Block 1 were associated with variation in HL and FY, with H2/H2 showing higher values for these traits. No significant associations with the five examined traits were detected for the Block 2 haplotype combinations. Haplotype combinations in Block 3 were associated with variation in SL, HL, and FY, with H10/H10 showing higher HL and FY values.

## 4. Discussion

Tianzhu White Yak fiber is a valuable textile raw material and has considerable potential for use in high-end textile products due to its unique morphological structure [31]. The coat of the Tianzhu White Yak is a mixed-type coat composed of multiple fiber types. Its intermediate fibers and coarse hairs can be processed into felt products, including linings, carpets, tents, and felt mats, and can also be used in overcoat fabrics [32]. In addition to providing economic value to herders through meat and milk production, Tianzhu White Yak also has high utilization value in terms of its fiber resources [6]. Therefore, characterizing the genetic basis of fiber production traits is important for the genetic improvement of Tianzhu White Yak. SNP analysis is widely used in livestock genetic research to identify molecular markers associated with economically important traits and to support marker-assisted selection [33]. Compared with other molecular markers, single nucleotide polymorphisms have the advantages of genetic stability, low detection cost, wide genomic coverage, high resolution, and high abundance [34]. The results of genetic diversity analysis showed that the PIC values of the selected SNP loci ranged from 0.25 to 0.50, indicating moderate polymorphism, and all loci conformed to Hardy–Weinberg equilibrium (*p* > 0.05). These results suggest that the selected loci contained moderate levels of genetic variation in the Tianzhu White Yak population and may provide useful information for subsequent association and marker evaluation [35].

Association analysis revealed consistent associations between genetic variants in *CFAP299* and *PRDM8* and fiber-related traits in Tianzhu White Yak. After multiple-testing correction, several loci also remained significantly associated with hair length at other body sites. These findings suggest that polymorphisms in these two genes may serve as candidate genetic markers for fiber-related traits. This result is consistent with previous genomic studies in Tianzhu White Yak. Liu et al. [6] identified *CFAP299* and *PRDM8* as candidate genes associated with hair length and fiber weight through GWAS. In addition, Bao et al. [4] identified a selection of loci on chromosome 6 that includes *FGF5* and *CFAP299*, further suggesting that this genomic region may be involved in variations in coat length. Lehman et al. [16] demonstrated that primary cilia are required for normal hair follicle morphogenesis in mice, while Dobbelaere et al. [17] identified *CFAP299* as a cilia-associated gene involved in ciliogenesis. These findings provide some support for the associations observed in this study. However, the specific roles of the *CFAP299* and *PRDM8* genes in hair growth remain unclear and require further functional studies. Associations between candidate gene polymorphisms and fiber traits have also been reported in other livestock species. Zhao et al. [36] found that several SNPs in the *FGF5* gene were associated with wool length and raw wool weight in fine-wooled sheep, suggesting their potential value as molecular markers. Therefore, the *CFAP299* and *PRDM8* polymorphisms identified in this study may serve as candidate markers for the genetic improvement of fiber-related traits in Tianzhu White Yak. However, all eight SNPs identified in this study are located in non-coding regions; therefore, they are considered candidate genetic markers. Although these loci may possess potential regulatory functions, current association analyses cannot confirm this. Further experiments—such as gene expression analysis and eQTL analysis—are required to determine whether these loci play a regulatory role.

Li et al. [37] found that overexpression of *CFAP299* inhibited apoptosis and promoted cell cycle progression in GC-1 spermatogonial cells, whereas siRNA-mediated knockdown of *CFAP299* increased apoptosis and caused cell cycle arrest at the G2/M phase, suggesting that this gene may be involved in the regulation of cell cycle progression and apoptosis. Although direct evidence linking *CFAP299* to hair follicle and skin development is currently limited, this gene encodes a cilia- and flagella-associated protein. Primary cilia are present in skin and hair follicle cells and play important roles in the hair follicle cycle and Hedgehog signaling transduction [16]. This study identified five SNP loci within the *CFAP299* gene that are significantly associated with head hair length and fiber yield in Tianzhu White Yak. These findings suggest that *CFAP299* may be a candidate gene associated with fiber-related traits in Tianzhu White Yak and may provide useful information for future molecular marker evaluation. In addition to *CFAP299*, polymorphisms in *PRDM8* were also significantly associated with hair-related traits in Tianzhu White Yak. *PRDM8* belongs to the PRDM gene family. Members of this family usually contain a PR domain and zinc finger motifs and are involved in the regulation of cell-state transitions during development and developmental signaling pathways [19]. Therefore, *PRDM8* has been reported to function as a regulatory molecule involved in cell proliferation, differentiation, and maturation [38]. Dan et al. [39] performed whole-genome resequencing of 14 goat populations and identified several genes, including *PRDM8*, which were suggested to play important roles in high-altitude adaptation, cashmere production, and responses to climatic variation in goats. In this study, following FDR correction, eight SNP loci within the *CFAP299* and *PRDM8* genes remained significantly associated with head hair length and fiber yield (*q* < 0.05). These results provide further genetic evidence supporting *CFAP299* and *PRDM8* as candidate genes for fiber-related traits; however, the present findings do not establish a direct regulatory role for these SNPs in fiber growth.

Linkage disequilibrium (LD) refers to the non-random association of alleles at two or more loci. Certain alleles are often inherited together in a non-random manner, resulting in haplotypes that occur at relatively high frequencies in a population and contributing to the observed LD [40]. The development of linkage disequilibrium maps and the characterization of haplotype blocks at the population level are useful for identifying candidate genes and genomic regions associated with phenotypic variation [41]. Qanbari [42] suggested that limited population size is an important factor contributing to LD, as the effective population size of most domestic animals has been substantially reduced. In the present study, LD analysis of the eight SNP loci in *CFAP299* and *PRDM8* identified three haplotype blocks, with strong LD among SNPs within each block. The observed LD pattern may partly reflect the demographic history and population structure of Tianzhu White Yak; however, this possibility was not directly evaluated in the present study. A haplotype refers to a combination of alleles located on the same chromosome and inherited together [43]. Since single-SNP analysis only reflects local information from an individual variant, and SNP loci may be jointly inherited through linkage disequilibrium, haplotype analysis can integrate information from multiple tightly linked SNPs and thereby complement single-locus association analysis [44,45]. Previous studies have shown that haplotype analysis can integrate information from multiple closely linked SNP loci, reflect the joint association patterns of multiple variants within the same genomic region, and be used to identify favorable haplotypes or haplotype combinations associated with economically important traits [46].

Further analysis of the relationships between different haplotype combinations and hair length traits and fiber yield in Tianzhu White Yak showed that, in Block 1, the H2/H2 combination had significantly higher HL and FY than some other haplotype combinations, suggesting that this combination may represent a haplotype combination associated with higher head hair length and fiber yield. No significant differences in hair length traits or fiber yield were observed among the haplotype combinations in Block 2, indicating that no significant associations were detected between Block 2 haplotype combinations and these traits. In Block 3, the H10/H10 combination showed higher HL and FY values, while the H12/H12 combination had significantly higher SL than the other combinations, indicating that haplotype combinations in Block 3 may be associated with some hair length traits and fiber yield in Tianzhu White Yak. These results suggest that haplotype combinations in Blocks 1 and 3 associated with higher phenotypic values may serve as candidate marker combinations for future marker-assisted selection, although their associations should be further validated in independent populations. Similar findings have also been reported in other fiber-producing livestock. Zhao et al. [47] found that two strongly linked haplotype blocks within the *KIF16B* gene were significantly associated with greasy fleece yield and wool length. Rong et al. [48] constructed 21 haplotype blocks and 68 haplotype combinations and found that multiple haplotype combinations were associated with cashmere yield, cashmere fineness, cashmere thickness, and cashmere length, suggesting that haplotype construction is useful for revealing the complex genetic variation underlying cashmere traits. Another study reported that SNP-derived haplotypes in the *FAT1* gene were significantly associated with mean wool fiber diameter, the standard deviation of wool fineness, and fiber diameter variation. In addition, a previous GWAS in Tianzhu White Yak identified a large number of SNPs on chromosome 6 that were significantly associated with fiber weight and head hair length, and these SNPs formed multiple linkage disequilibrium blocks, indirectly indicating that fiber-related traits in Tianzhu White Yak may be jointly influenced by multiple SNP loci [6]. Gabriel et al. [49] suggested that haplotype blocks may result from the combined effects of several mechanisms, including domestication, population subdivision, founder events, selection, and recombination hotspots. Such extended linkage disequilibrium blocks are frequently observed in livestock populations. These population genetic processes may also have contributed to the haplotype structure observed among the eight SNP loci in *CFAP299* and *PRDM8*.

## 5. Limitations

We acknowledge several limitations of this study. First, there was a gender imbalance in the study population, with 564 females and 195 males. Although gender and age were included as fixed effects in the association model, the small sample size of males may have reduced the precision of estimates for males and did not fully rule out the influence of sexual dimorphism on body size and coat-related traits. Second, this study was based on genetic association analysis and did not include functional validation of *CFAP299* or *PRDM8*. Finally, the identified single-nucleotide polymorphisms (SNPs) are located in non-coding regions, and no gene expression assays in hair follicles or other functional assays were conducted. Therefore, these SNPs should be considered candidate markers. Future studies should utilize more representative sample populations and incorporate gene expression and functional validation studies to elucidate the biological effects of these loci.

## 6. Conclusions

This study identified significant associations between polymorphisms in *CFAP299* and *PRDM8* and fiber-related traits in Tianzhu White Yak. After adjustment for sex and age and FDR correction, all eight SNP loci were associated with head hair length and fiber yield, while several loci were also associated with other hair length traits. Individuals carrying mutant homozygous genotypes generally showed favorable performance in fiber yield and some hair length traits. Linkage disequilibrium and haplotype analyses identified three haplotype blocks. The H2/H2 combination in Block 1 was associated with higher HL and FY, while the H10/H10 combination in Block 3 was associated with higher HL and FY, and the H12/H12 combination showed higher SL. These polymorphisms and haplotype combinations may serve as candidate genetic markers for fiber traits in Tianzhu White Yak and could potentially support future marker-assisted selection after validation in independent populations and functional studies.

## Figures and Tables

**Table 1:** SNP site primer information.

Genes Name	SNPs Name	Primer Sequences (5′ → 3′)	Length/bp	Annealing Temperature/°C
	g.24886108 A > T	F: AAAGTTTTCCGCCCCCTGG R: CCCTCTATCCTCCTGTCCTCA	373	58
	g.24928028 C > T	F: CCATGGGGTCTCGAAGAGTT R: TCCATGAACAGAGGAGCTGG	357	59.5
*CFAP299*	g.25200739 G > A	F: AGGATGTCTGGCTCTAGGTGA R: TGCTCTGGTCATAGGCTCACA	556	60
	g.25210441 C > T	F: TTGAACCCATGTCTCTCGCA R: ACAGGACTAGAGTAAGCAGGGA	480	59
	g.25237572 A > C	F: GACCCACGTTTGATCCTTGG R: CTGGGTCTCCTGCGTTGTAA	416	59.5
	g.25499166 G > A	F: TCCCAGGTGGTACAGTTGGT R: GTGAGTCGTCCCTGTTGTGG	374	58
*PRDM8*	g.25571255 G > T	F: GTGTGAAAGTCAATGGAAAGTCC R: AGCAGGTGAGTGACAAGCAT	524	56
	g.25622784 C > T	F: CCAACAGTCACAGCTACAGAGG R: AGAGGCAGTGTGGCTAAGGA	393	59

**Table 2:** Descriptive statistics on fiber yield and hair length traits of Tianzhu White Yak.

Trait	Mean	Max	Min	Standard Error
FY (kg)	1.43	3.83	0.26	0.02
SL (cm)	26.63	57	12	0.14
BSL (cm)	17.38	32	3	0.25
HL (cm)	11.80	27	3	0.14
BL (cm)	17.09	40	3	0.29

Note: Fiber yield (FY), skirt hair length (SL), body side hair length (BSL), head hair length (HL), and back hair length (BL).

**Table 3:** Variation information and diversity parameters for SNP loci in the *CFAP299* and *PRDM8* genes.

Genes	SNPs	Position	Genotypic Frequencies			Allelic Frequencies		MAF	He	Ne	PIC	χ^2^	*p*-Value
*CFAP299*	g.24886108 A > T	Intron	AA	AT	TT	A	T	0.376	0.469	1.884	0.359	3.803	0.051
			0.406	0.436	0.158	0.624	0.376						
	g.24928028 C > T	Intron	CC	CT	TT	C	T	0.389	0.476	1.907	0.362	1.474	0.225
			0.383	0.455	0.162	0.611	0.389						
	g.25200739 G > A	Intron	GG	GA	AA	G	A	0.480	0.499	1.997	0.375	0.125	0.723
			0.233	0.493	0.274	0.480	0.521						
	g.25210441 C > T	Intron	CC	CT	TT	C	T	0.448	0.494	1.978	0.372	0.010	0.919
			0.306	0.493	0.201	0.553	0.448						
	g.25237572 A > C	Intron	AA	AC	CC	A	C	0.426	0.489	1.957	0.369	0.577	0.447
			0.336	0.476	0.188	0.574	0.426						
*PRDM8*	g.25499166 G > A	Intron	GG	GA	AA	G	A	0.289	0.411	1.698	0.327	0.008	0.927
			0.506	0.410	0.084	0.711	0.289						
	g.25571255 G > T	upstream	GG	GT	TT	G	T	0.397	0.479	1.919	0.364	0.072	0.789
			0.361	0.484	0.155	0.603	0.397						
	g.25622784 C > T	upstream	CC	CT	TT	C	T	0.333	0.444	1.799	0.346	1.568	0.210
			0.455	0.424	0.121	0.667	0.333						

**Table 4:** Correlation analysis between the *CFAP299* gene variants g.24886108 A > T, g.24928028 C > T, g.25200739 G > A, g.25210441 C > T, and g.25237572 A > C in Tianzhu White Yak and traits related to fiber yield and hair length.

Genotype	SL/cm	BSL/cm	HL/cm	BL/cm	FY/kg
SNPs g.24886108 A > T					
AA	26.15 ± 0.29	16.99 ± 0.48 ^b^	10.71 ± 0.26 ^c^	21.02 ± 0.42	1.27 ± 0.04 ^c^
AT	26.52 ± 0.29	17.75 ± 0.47 ^ab^	12.15 ± 0.25 ^b^	21.97 ± 0.41	1.41 ± 0.04 ^b^
TT	25.81 ± 0.46	19.21 ± 0.77 ^a^	13.35 ± 0.41 ^a^	22.33 ± 0.68	1.78 ± 0.07 ^a^
*p*-Value	*p* = 0.348	*p* = 0.062	*p* < 0.001	*p* =0.148	*p* < 0.001
*q*-Value	*q* = 0.387	*q* = 0.092	*q* < 0.001	*q* =0.179	*q* < 0.001
SNPs g.24928028 C > T					
CC	26.14 ± 0.30	17.09 ± 0.50	10.52 ± 0.27 ^c^	20.92 ± 0.44	1.26 ± 0.04 ^c^
CT	26.45 ± 0.28	17.67 ± 0.46	12.16 ± 0.25 ^b^	21.99 ± 0.40	1.40 ± 0.04 ^b^
TT	25.98 ± 0.45	18.99 ± 0.75	13.45 ± 0.40 ^a^	22.31 ± 0.66	1.77 ± 0.07 ^a^
*p*-Value	*p* = 0.560	*p* = 0.143	*p* < 0.001	*p* = 0.101	*p* < 0.001
*q*-Value	*q =* 0.590	*q =* 0.178	*q <* 0.001	*q =* 0.130	*q <* 0.001
SNPs g.25200739 G > A					
GG	26.54 ± 0.36	16.53 ± 0.59 ^b^	10.19 ± 0.31 ^c^	20.18 ± 0.52 ^b^	1.16 ± 0.05 ^c^
GA	26.37 ± 0.27	17.46 ± 0.45 ^b^	11.48 ± 0.23 ^b^	21.81 ± 0.39 ^a^	1.37 ± 0.04 ^b^
AA	25.72 ± 0.37	19.30 ± 0.60 ^a^	13.99 ± 0.31 ^a^	22.86 ± 0.52 ^a^	1.74 ± 0.05 ^a^
*p*-Value	*p* = 0.191	*p* = 0.002	*p* < 0.001	*p* < 0.001	*p* < 0.001
*q*-Value	*q =* 0.218	*q =* 0.004	*q <* 0.001	*q =* 0.001	*q <* 0.001
SNPs g.25210441 C > T					
CC	26.57 ± 0.32	16.72 ± 0.53 ^b^	10.25 ± 0.27 ^c^	20.63 ± 0.46 ^b^	1.18 ± 0.05 ^c^
CT	26.31 ± 0.27	17.76 ± 0.45 ^ab^	11.75 ± 0.23 ^b^	21.92 ± 0.39 ^ab^	1.40 ± 0.04 ^b^
TT	25.46 ± 0.44	19.31 ± 0.72 ^a^	14.75 ± 0.36 ^a^	22.81 ± 0.63 ^a^	1.91 ± 0.06 ^a^
*p*-Value	*p* = 0.098	*p* = 0.010	*p* < 0.001	*p* = 0.008	*p* < 0.001
*q*-Value	*q =* 0.130	*q =* 0.017	*q* < 0.001	*q =* 0.015	*q* < 0.001
SNPs g.25237572 A > C					
AA	26.17 ± 0.32	16.72 ± 0.52 ^b^	10.27 ± 0.26 ^c^	20.19 ± 0.45 ^b^	1.21 ± 0.04 ^c^
AC	26.38 ± 0.27	17.71 ± 0.45 ^ab^	11.68 ± 0.22 ^b^	22.26 ± 0.39 ^a^	1.37 ± 0.04 ^b^
CC	26.08 ± 0.44	19.48 ± 0.73 ^a^	15.01 ± 0.36 ^a^	22.81 ± 0.63 ^a^	1.92 ± 0.06 ^a^
*p*-Value	*p* = 0.773	*p* = 0.006	*p* < 0.001	*p* < 0.001	*p* < 0.001
*q*-Value	*q =* 0.773	*q =* 0.011	*q* < 0.001	*q* < 0.001	*q* < 0.001

Note: Values are presented as least-squares means ± standard error (LS-means ± SE). Different lowercase superscript letters within the same SNP locus and trait indicate significant differences among genotypes according to Tukey’s multiple comparison test (*p* < 0.05), whereas genotypes sharing at least one lowercase letter do not differ significantly. FDR-adjusted *q*-values were calculated using the Benjamini–Hochberg method across all 40 SNP–trait tests, and *q* < 0.05 was considered statistically significant.

**Table 5:** Correlation analysis between the *PRDM8* gene variants g.25499166 G > A, g.25571255 G > T, and g.25622784 C > T in Tianzhu White Yak and traits related to fiber yield and hair length.

Genotype	SL/cm	BSL/cm	HL/cm	BL/cm	FY/kg
SNPs g.25499166 G > A					
GG	26.49 ± 0.27	17.19 ± 0.45	10.84 ± 0.23 ^c^	20.94 ± 0.39 ^b^	1.26 ± 0.04 ^c^
GA	26.05 ± 0.29	17.85 ± 0.48	12.18 ± 0.25 ^b^	22.12 ± 0.42 ^ab^	1.47 ± 0.04 ^b^
AA	25.90 ± 0.64	19.70 ± 1.05	15.23 ± 0.55 ^a^	23.37 ± 0.91 ^a^	1.97 ± 0.09 ^a^
*p*-Value	*p* = 0.438	*p* = 0.087	*p* < 0.001	*p* = 0.014	*p* < 0.001
*q*-Value	*q =* 0.474	*q =* 0.120	*q* < 0.001	*q =* 0.023	*q* < 0.001
SNPs g.25571255 G > T					
GG	26.45 ± 0.31	17.03 ± 0.52 ^b^	10.59 ± 0.27 ^c^	20.81 ± 0.45 ^b^	1.24 ± 0.05 ^c^
GT	25.88 ± 0.27	17.59 ± 0.45 ^ab^	11.81 ± 0.23 ^b^	21.75 ± 0.39 ^ab^	1.44 ± 0.04 ^b^
TT	26.97 ± 0.45	19.40 ± 0.74 ^a^	14.29 ± 0.39 ^a^	23.18 ± 0.65 ^a^	1.70 ± 0.07 ^a^
*p*-Value	*p* = 0.077	*p* = 0.028	*p* < 0.001	*p =* 0.006	*p* < 0.001
*q*-Value	*q =* 0.110	*q =* 0.044	*q* < 0.001	*q =* 0.011	*q* < 0.001
SNPs g.25622784 C > T					
CC	26.43 ± 0.28	17.20 ± 0.47	10.72 ± 0.24 ^c^	20.90 ± 0.41	1.28 ± 0.04 ^c^
CT	26.06 ± 0.28	17.80 ± 0.47	12.09 ± 0.24 ^b^	22.10 ± 0.41	1.43 ± 0.04 ^b^
TT	26.34 ± 0.54	19.08 ± 0.90	14.67 ± 0.47 ^a^	22.74 ± 0.78	1.86 ± 0.08 ^a^
*p*-Value	*p* = 0.664	*p* = 0.185	*p* < 0.001	*p* = 0.032	*p* < 0.001
*q*-Value	*q =* 0.681	*q =* 0.218	*q* < 0.001	*q =* 0.049	*q* < 0.001

Note: Values are presented as least-squares means ± standard error (LS-means ± SE). Different lowercase superscript letters within the same SNP locus and trait indicate significant differences among genotypes according to Tukey’s multiple comparison test (*p* < 0.05), whereas genotypes sharing at least one lowercase letter do not differ significantly. FDR-adjusted *q*-values were calculated using the Benjamini–Hochberg method across all 40 SNP–trait tests, and *q* < 0.05 was considered statistically significant.

**Table 6:** Haplotype combination types and frequencies of SNP loci in the *CFAP299* and *PRDM8* genes.

Block	Haplotype Combinations	Number of Yaks	Frequencies
	H1/H2 (AC/TT)	323	0.426
	H1/H1 (AC/AC)	287	0.378
	H2/H2 (TT/TT)	118	0.155
Block 1	H1/H3 (AC/AT)	18	0.024
	H1/H4 (AC/TC)	4	0.005
	H3/H2 (AT/TT)	4	0.005
	H4/H2 (TC/TT)	4	0.005
	H3/H3 (AT/AT)	1	0.002
	H6/H5 (AT/GC)	323	0.426
	H5/H5 (GC/GC)	174	0.229
	H6/H6 (AT/AT)	150	0.198
Block 2	H8/H6 (AC/AT)	48	0.063
	H8/H5 (AC/GC)	48	0.063
	H8/H8 (AC/AC)	10	0.013
	H6/H7 (AT/GT)	3	0.004
	H5/H7 (GC/GT)	3	0.004
	H9/H9 (GG/GG)	269	0.354
	H10/H9 (AT/GG)	261	0.344
	H9/H12 (GG/GT)	103	0.136
	H10/H10 (AT/AT)	61	0.080
Block 3	H10H12 (AT/GT)	45	0.059
	H12/H12 (GT/GT)	12	0.016
	H11/H9 (AG/GG)	5	0.007
	H11/H10 (AG/AT)	3	0.004

## Data Availability

Genotypic data for the 8 SNP loci in the *CFAP299* and *PRDM8* genes, corresponding phenotypic records for 759 Tianzhu White Yaks, haplotype combinatorial association analysis results, and representative Sanger sequencing files for each SNP locus are all available in the Zenodo database at https://doi.org/10.5281/zenodo.20441515. Of the 759 Tianzhu White Yaks analyzed in this study, the original whole-genome resequencing data for 758 individuals are publicly available in the NCBI Sequence Read Archive (SRA), project number PRJNA1521123. Due to corrupted original FASTQ files and the lack of complete backups, the original sequencing data for individuals 3–69 cannot be submitted.

## Supplementary Materials

The following supporting information can be downloaded at: https://www.mdpi.com/article/10.3390/life16091559/s1, Supplementary Table S1. Association analysis between haplotype combinations of different blocks in the *CFAP299* and *PRDM8* genes and hair length traits and fiber yield in Tianzhu White Yak.; Supplementary Table S2. Genotype data; Supplementary Table S3. Phenotypic data.

## Abbreviations

The following abbreviations are used in this manuscript:

LD	Linkage disequilibrium
SNP	Single nucleotide polymorphism
